# Supplementary material for: Is the bispectral index monitoring protective against postoperative cognitive decline? A systematic review with meta-analysis
Source: PLoS One. 2020 Feb 13;15(2):e0229018. doi: 10.1371/journal.pone.0229018 (PMC7018011; doi:10.1371/journal.pone.0229018)
Supplement: S1 Table — (DOCX) [file pone.0229018.s001.docx]

**S 1 Table. Characteristics of the studies excluded.**

| Authors (year, country) | Year of recruitment | Form | Study design | Reason for exclusion |
| --- | --- | --- | --- | --- |
| Rohm *et al.* (2005, Germany)[40] | Not stated | RCT | Bispectral index (BIS)‐controlled desflurane-fentanil (n=24) or propofol/remifentanil (*n*=25) anaesthesia undergoing elective abdominal prostatectomy and there are with neuropsychological test | Retracted article |
| Erdogan *et al.* (2012, Turkey)[41] | 2007–2008 | RCT | Propofol-remifentanil anaesthesia with BIS-monitoring (group1 n=45, group2 n=47) undergoing elective orthopaedic procedures and there are with pre-, and postoperative neuropsychological test | Randomisation was by pre-operative MMSE |
| Deiner *et al.* (2015, USA)[42] | Not stated | nRT | Anaesthesia with BIS monitoring (n=105) of major non-cardiac surgery undergoing general anaesthesia including general, spine, urologic, or thoracic surgery and there are with pre-, and postoperative neuropsychological tests | Insufficient data and nRT |
| Egawa *et al.* (2015, Japan)[43] | Not stated | RCT | Propofol (n=60) or sevoflurane (n=58) anaesthesia with BIS monitoring undergoing elective lung surgery and there are with pre-, and postoperative neuropsychological tests | Randomisation was by anaesthetic agent |
| Ishida *et al.* (2015, Japan)[44] | Not stated | nRT | Anaesthesia (n=62) undergoing CABG with BIS monitoring and there are with pre-, and postoperative tests | Insufficient data and nRT |
| Zhang and Nie (2016, China)[45] | Not stated | RCT | TIVA with remifentanil and propofol given by target-controlled infusion on postoperative cognitive function in young and middle-aged patients undergoing gynaecological laparoscopic surgery and there are with pre-, and postoperative neuropsychological tests | Insufficient data and nRT |
| Cao *et al.* (2017, China)[46] | 2014–2015 | nRT | BIS-guided (45–55) (n=33) or no BIS-guided propofol-sufentanil anaesthesia (n=27) undergoing liver transplantation and there are with pre-, and postoperative neuropsychological tests | nRT |
| Cotoia *et al.* (2017, Italy)[47] | 2014–2016 | RCT | AutoTIVA (n=30) or desflurane (n=30) or sevoflurane (n=30) or manual TIVA (n=30) anaesthesia with BIS-monitoring undergoing elective urologic surgery and there are with pre-, and postoperative neuropsychological test | Randomisation was by anaesthetic agent |
| RCT: randomised controlled trial; nRT: non-randomised controlled trial; BIS: bispectral index; MMSE: Mini Mental State Examination; CABG: Coronary Artery Bypass Grafting; TIVA: Total Intravenous Anaesthesia. | | | | |
