# Supplementary material for: Is the bispectral index monitoring protective against postoperative cognitive decline? A systematic review with meta-analysis
Source: PLoS One. 2020 Feb 13;15(2):e0229018. doi: 10.1371/journal.pone.0229018 (PMC7018011; doi:10.1371/journal.pone.0229018)
Supplement: S1 Appendix — (DOCX) [file pone.0229018.s004.docx]

**S1 Appendix. Methodological details.**

Search and data extraction

Our query in MEDLINE combined Medical Subject Headings and free-text terms, as follows: (*POD OR POCD OR ((postoperative OR perioperative) AND delirium[MeSH]) OR ((“perioperative period”[MeSH] OR “postoperative period” [MeSH]) AND (“cognitive function” OR “cognitive dysfunction”[MeSH] OR “cognitive impairment” OR “cognitive decline” OR “cognitive deficit” OR “cognitive outcome”)) AND (BIS OR “bispectral index” OR “bispectral monitoring” OR “depth of anaesthesia” OR “depth of anesthesia” OR “consciousness monitors” [MeSH])*.

After combining the records with reference manager software (EndNote X7.4, Clarivate Analytics, Philadelphia, PA, US), overlaps between databases and duplicates were removed. Then, two authors (TB, MK) selected the articles by title, abstract, and full-text in duplicate; discrepancies were resolved by consensus.

Data were extracted by review authors (TB, MK) in duplicate as follows: publication data (author, year and reference), study design and arms (with the number and characteristics of patients and BIS monitoring), type and definition of postoperative cognitive dysfunction and cognitive tests (types and before-and-after values with corresponding probability values).

Protocol deviations

Since we improved the coverage of the search, we re-designed the query and updated the search. Although we planned to pool neuropsychological test results as well, the discrepancy of the tests applied and the reporting of the results did not allow us to do so. Similarly, the study groups were re-defined based on the timing of the outcome measurements to obtain RRs. To improve the quality of evidence, we decided to include RCTs exclusively.
