# Supplementary material for: Is the bispectral index monitoring protective against postoperative cognitive decline? A systematic review with meta-analysis
Source: PLoS One. 2020 Feb 13;15(2):e0229018. doi: 10.1371/journal.pone.0229018 (PMC7018011; doi:10.1371/journal.pone.0229018)
Supplement: Attachment — Submitted filename: BIS paper .docx [file pone.0229018.s006.docx]

The authors are to be commended for a rigorous attempt to answer 2 questions...does BIS monitoring affect the development of POD or POCD and if the occurrence of a high or low BIS number affects the development of these complications. In an analysis of 1985 patients the conclusions drawn suggest that BIS monitoring is protective of POD on day 1 and protective of POCD at week 12. The level of BIS, high or low did not make a difference in the anlaysis. However, I do not believe that these conclusions can be drawn based on several factors that are not identified; age must be considered, type of anesthetic used, duration of anesthesia, type of surgery preoperative condition, preemptive measures against POD, postoperative complications such as pneumonia, infection or fever, and importantly, what if any measures were taken based on BIS monitoring. For example, if the BIS was low, was the degree of sedation reduced?

Several other studies have looked at BIS monitoring. Orena et al concluded that:

*“Use of a depth of anesthesia monitor and lighter sedation had the strongest evidence in reduction of POD. Perioperative dexmedetomidine, ketamine, dexamethasone, and antipsychotic administration may reduce the risk of POD” .(*[Orena EF](https://www.ncbi.nlm.nih.gov/pubmed/?term=Orena%20EF%5BAuthor%5D&cauthor=true&cauthor_uid=26822815) et al The role of anesthesia in the prevention of postoperative delirium: a systematic review [Minerva Anestesiol.](https://www.ncbi.nlm.nih.gov/pubmed/26822815) 2016;82(6):669-83.) Other measures were probably also effective

Choi et al developed a screening tool that “*successfully identified patients at a high risk of POD at admission. The POD prevention project was feasible to implement, effective in preventing delirium, and improved knowledge regarding delirium among the medical staff”* (Choi et al . Impact of a delirium prevention project among older hospitalized patients who underwent orthopedic surgery: a retrospective cohort study [BMC Geriatr.](https://www.ncbi.nlm.nih.gov/pubmed/31655551) 2019 Oct 26;19(1):289. doi: 10.1186/s12877-019-1303-z. The emphasis here was on geriatric patients.

Lee et al also noted that POD is a risk factor for later dementia and following a metanalysis concluded that, “*POD after hip surgery is a risk factor for incident dementia. Early identification of cognitive function should be needed after surgery and appropriate prevention and treatment for dementia will be required, especially in cases with POD.”* [Lee SJ](https://www.ncbi.nlm.nih.gov/pubmed/?term=Lee%20SJ%5BAuthor%5D&cauthor=true&cauthor_uid=31751902), et al Postoperative delirium after hip surgery is a potential risk factor for incident dementia: A systematic review and meta-analysis of prospective studies. [Arch Gerontol Geriatr.](https://www.ncbi.nlm.nih.gov/pubmed/31751902) 2019 Nov 11;87:103977. doi: 10.1016/j.archger.2019.103977. [Epub ahead of print]. Preoperative recognition of dementia should be made and appropriate measures taken such as medication adjustment.

Specific to this manuscript are the following:

1. All the requirements of PLOS One have been met with the exception of the conclusions drawn
2. The paper could be shortened by eliminating explanations of studies not considered relevant. In general it could be simplified.
3. Any reasons suggested for why neither low nor high BIS values impacted POD at day 1 (In contradiction to other studies)?
4. Lines 91-93 are confusing: l_1_ vs C_1…_is that the same as I^1^ vs Chi^1^ ?
5. Please reconcile the statements “narcotics do not affect POD and POCD” and “deep anesthesia may”?
6. Of the 14 authors, only one appears to be an anesthesiologist. Given that anesthesia has been implicated in POD and POCD, more impute should be provided from this specialty.
7. The authors note that 6461 patients would be required for a final conclusion and given that only 1/3 are included in this analysis, then even a suggested association with BIS monitoring and POD or POCD may not be warranted. Certainly, more detailed and larger studies looking at many more factors are necessary. Given that some decades ago something akin to mass hysteria arose insisting that BIS monitoring should be the standard of care .Medico legal implications resulted. Subsequent studies have disapproved this insistence. Until more is understood about the development especially of POCD in the elderly, universal BIS monitoring should not be even considered as essentil. It may be a tool, but there are too many unanswered questions. And there are other entropy monitors.

.
